# Adherent-invasive *Escherichia coli*, strain LF82 disrupts apical junctional complexes in polarized epithelia

**DOI:** 10.1186/1471-2180-9-180

**Published:** 2009-08-26

**Authors:** Eytan Wine, Juan C Ossa, Scott D Gray-Owen, Philip M Sherman

**Affiliations:** 1 Research Institute, Hospital for Sick Children, 555 University Avenue, Toronto, Ontario, M5G 1X8, Canada; 2 Department of Molecular Genetics, University of Toronto, 1 King's College Circle, Toronto, Ontario, M5S 1A8, Canada; 3 Department of Pediatrics, Division of Gastroenterology and Nutrition, University of Alberta, 11402 University Avenue, Edmonton, Alberta, T6G 2J3, Canada

## Abstract

**Background:**

Although bacteria are implicated in the pathogenesis of chronic inflammatory bowel diseases (IBD), mechanisms of intestinal injury and immune activation remain unclear. Identification of adherent-invasive *Escherichia coli *(AIEC) strains in IBD patients offers an opportunity to characterize the pathogenesis of microbial-induced intestinal inflammation in IBD. Previous studies have focused on the invasive phenotype of AIEC and the ability to replicate and survive in phagocytes. However, the precise mechanisms by which these newly identified microbes penetrate the epithelial lining remain to be clarified. Therefore, the aim of this study was to delineate the effects of AIEC, strain LF82 (serotype O83:H1) on model polarized epithelial monolayers as a contributor to intestinal injury in IBD.

**Results:**

Infection of T84 and Madin-Darby Canine Kidney-I polarized epithelial cell monolayers with AIEC, strain LF82 led to a reduction in transepithelial electrical resistance and increased macromolecular (10 kilodalton dextran) flux. Basolateral AIEC infection resulted in more severe disruption of the epithelial barrier. Increased permeability was accompanied by a redistribution of the tight junction adaptor protein, zonula occludens-1, demonstrated by confocal microscopy and formation of gaps between cells, as shown by transmission electron microscopy. After 4 h of infection of intestine 407 cells, bacteria replicated in the cell cytoplasm and were enclosed in membrane-bound vesicles positive for the late endosomal marker, LAMP1.

**Conclusion:**

These findings indicate that AIEC, strain LF82 disrupts the integrity of the polarized epithelial cell barrier. This disruption enables bacteria to penetrate into the epithelium and replicate in the host cell cytoplasm. These findings provide important links between microbes related to IBD, the intestinal epithelial cell barrier and disease pathogenesis.

## Background

The inflammatory bowel diseases (IBD), Crohn disease and ulcerative colitis, are relatively common chronic disorders considered to develop due to an aberrant immune response to intestinal microbes in a genetically susceptible host [1]. Human data and murine models both implicate the involvement of luminal bacteria in IBD pathogenesis. For example, inflammation is induced by direct delivery of fecal material into non-inflamed bowel loops in susceptible individuals [2] and diversion of feces results in distal improvement in mucosal inflammation [3]. In addition, most of the genes associated with susceptibility to IBD, including NOD2/CARD15, Atg16L1 and IRGM encode proteins involved in host-microbial interactions [4]. Further support for the involvement of microbes in the pathogenesis of IBD is based on the observation that colitis does not occur in most gene knock-out models of IBD when animals are reared in germ-free conditions [5,6].

Recent advances in molecular techniques have identified a reduction in the phyla Firmicutes and Bacteroidetes in IBD patients [7]. Although several organisms have been proposed as a cause of IBD, there is still no compelling evidence that any one specific microbe is the etiologic agent. *Mycobacterium avium *subspecies paratuberculosis has been suggested by some investigators [8], although this remains an area of ongoing controversy [9]. Most recently, absence of *Faecalibacterium prausnitzii *from the ileum of patients with Crohn disease undergoing surgical resection was associated with recurrence of disease, suggesting a protective role for this commensal organism [10].

Observations linking IBD to an increase in adherent *Escherichia coli *strains have also been recognized over the past decade [11]. Invasive properties of some of these isolates, including *E. coli *strain LF82 (serotype O83:H1), led to the proposition that adherent-invasive *E. coli *strains (also termed AIEC) are involved in disease pathogenesis [12]. Such an association is supported by the isolation of AIEC from 36% of ileal lesions in post-surgical resection Crohn disease patients, compared to just 6% of healthy controls [13], accompanied by increased prevalence and diversity of AIEC strains in patients with Crohn disease [14]. Although some of the mechanisms by which these bacteria lead to colonization and intestinal injury, such as induction of carcinoembryonic antigen-related cell-adhesion molecule (CEACAM)-6 receptor expression by TNF-α [15], have been well characterized, other virulence traits remain to be determined.

Defects in the structure and function of apical junctional complexes (AJCs) are implicated in both patients with IBD and in animal models of IBD [16,17]. In this context, the adverse effects of microbes on intercellular junctions offer potential bridges connecting bacteria to the pathogenesis of IBD. Barrier dysfunction precedes the relapse of Crohn disease in asymptomatic patients [18] and is also seen in unaffected first-degree relatives, who are at increased risk of subsequently developing the illness [19]. Recent studies demonstrate specific distribution patterns of the tight junction proteins claudin 2, 3, 4, 5, & 8 in IBD patients, which correlate with increased gut permeability [20,21]. For these reasons, the aim of this study was to define the ability of AIEC strain LF82 to disrupt model epithelial cell polarized monolayers. We describe herein increased permeability of polarized epithelia infected with AIEC as well as morphologic disruption of apical junction complexes.

## Methods

### Epithelial cells in tissue culture

T84 and Madin-Darby Canine Kidney (MDCK)-I cells are polarized epithelial cells that form AJCs, resulting in high electrical resistance, and are widely used for studying the effects of bacteria on permeability [22,23]. T84 human colon cancer epithelial cells were cultured in Dulbecco's minimal essential medium (DMEM)/F-12, 10% heat-inactivated fetal bovine serum (FBS), 2% penicillin-streptomycin, 2% sodium bicarbonate and 0.6% L-glutamine. MDCK-I cells were grown in DMEM, 10% FBS and 2% penicillin-streptomycin (all from Gibco, Grand Island, NY). Cells were maintained in 25 cm^2 ^flasks (Corning Glass Works, Corning, NY) and then grown on 12-well Transwells (6.5 mm diameter; 3 μm pore size; 37°C; 5% CO_2_; Corning) or 24-well plates (Corning). Non-polarized intestine 407 (human fetal intestine) cells were cultivated in Minimal Essential Medium (MEM), 10% FBS and 2% penicillin-streptomycin (Gibco).

### Bacterial strains

Enterohemorrhagic *E. coli *(EHEC), strain CL56 serotype O157:H7 [24], non-pathogenic *E. coli*, laboratory strain HB101, used as a negative control, and adherent-invasive *E. coli *(AIEC), strain LF82 serotype O83:H1, a generous gift from Dr. Darfeuille-Michaud (Université d'Auvergne, Clermont-Ferrand, France) [13] were stored at -80°C and re-grown on 5% sheep blood agar plates at 37°C. Colonies were transferred from plates into Penassay broth and incubated at 37°C for 18 h, and re-grown in 10:1 fresh Penassay broth (3 h; 37°C). Multiplicity of infection (MOI) used for all experiments was 100:1. To determine whether live bacteria were required for the observed effects, bacterial suspensions were either boiled at 100°C for 30 min or fixed with formaldehyde for 6 h prior to infection of cell monolayers.

### Measurement of transepithelial electrical resistance (TER) and macromolecular permeability

MDCK-I and T84 cells were plated onto Transwells (5 × 10^4 ^or 2 × 10^5 ^cells/well, respectively; 6.5 mm diameter; 0.4 μm-pore size; Corning) and grown until AJCs developed (as indicated by a TER > 1,000 Ω·cm^2^). Twenty four hours prior to infection the tissue culture medium was removed and fresh medium without antibiotics, but with FBS, was added. FBS was maintained throughout the infection period. Transwells were then infected with either EHEC O157:H7, *E. coli *HB101 or AIEC (MOI: 100:1; 37°C; 5% CO_2_) introduced either to the apical or basolateral aspect of the Transwell. Sham control monolayers were treated in an identical fashion, excluding the addition of bacteria. TER was measured prior to and 16 h after infection, using a Millicell-ERS Voltmeter and chopstick electrodes (Millpore, Bedford, MA). TER of Transwells without cells was 32 Ω·cm^2^. Results are expressed as a percentage, relative to sham control wells.

Dextran flux was used to measure paracellular macromolecular permeability [25]. After 16 h of infection, monolayers were washed four times with phosphate-buffered saline (PBS) and infrared-labeled dextran (10-kDa; 0.2 ml of 0.1 mg/ml in DMEM; Alexa-Fluor 647, Molecular Probes, Eugene, OR) was then inserted into the apical compartment of Transwells. After 5 h at 37°C, the basal compartment was sampled, diluted 1:20, and loaded into 96-well plates for infrared signal quantification using an imaging system at 700 nm (Odyssey^®^, Licor, Rockford, IL). Integrated intensities were expressed relative to sham control polarized monolayers.

### Confocal microscopy for zonula occludens-1 (ZO-1) and lysosomal-associated membrane protein (LAMP)-1

For ZO-1 staining, MDCK-I cell monolayers were grown to confluence (TER >1,000 Ω·cm^2^) on 6.5 mm Transwells and then infected with AIEC, strain LF82 at a MOI of 100:1 for 16 h at 37°C. Monolayers were then washed 4 times with PBS and fixed with 100% cold methanol for 10 min at 4°C, blocked with 5% skim milk (Santa Cruz Biotechnology, Santa Cruz, CA) for 1 h at room temperature and then incubated with primary rabbit anti-ZO-1 antibodies (1:50, Zymed, Burlington, Ontario, Canada) for 1 h at room temperature. After rinsing 3 times for 10 min with PBS, cell monolayers were incubated with secondary antibodies, Cy2-goat anti-rabbit (1:200, Zymed), for 1 h at 20°C. After two further washes, 300 nM of 4',6-diamidino-2-phenylindole (DAPI, 1:36,000, Invitrogen, Eugene, ON) was added for 5 min, and rinsed off twice. Membranes supporting the monolayers were then excised and mounted onto glass slides (using DakoCytomation Mounting Medium, Carpentaria, CA).

For LAMP1 staining, intestine 407 cells were grown on glass cover slips in 24-well plates overnight and then either left uninfected or infected with AIEC, strain LF82 for 4 h at 37°C (MOI 100:1). Wells were washed 3 times with PBS (pH 7.0) and fixed with 4% paraformaldehyde in PBS for 20 min at 20°C. Wells were then washed with PBS and permeabilized with Triton-X 100 (0.1% in PBS; 20 min at 20°C) and blocked overnight with 5% skim milk (Santa Cruz) at 4°C. Wells were incubated with mouse monoclonal anti-LAMP1 antibodies (1 in 1,000 dilution; Developmental Studies Hybridoma Bank, Iowa City, IA) for 1 h at 20°C, washed 5 times in PBS and then incubated with secondary antibody, Cy3-goat anti-mouse (1:100, Zymed) for 1 h at 20°C. DAPI staining was performed, as detailed above, and coverslips mounted onto glass slides.

All samples were examined using a Leica DMIRE2 Quorum spinning disk confocal scan head inverted fluorescence microscope (Wetzlar, Germany), equipped with a Hamamatsu Back-Thinned EM-CCD camera (Hamamatsu, Japan), at 63× objective. Images were acquired and analyzed using Velocity 3.7.0 acquisition software (Improvision, Coventry, England).

### Transmission electron microscopy

Confluent MDCK-I Transwells were left uninfected or infected with AIEC, strain LF82 (MOI: 100:1; 4 h or 48 h; 37°C). Support membranes were washed, excised and cells fixed in formaldehyde (4%) and glutaraldehyde (1%) in phosphate buffer, and then post-fixed in osmium tetroxide (1%; 2 h; 20°C). Specimens were dehydrated in a graded series of acetone, and subsequently infiltrated and embedded in Epon-Araldite epoxy resin. The processing steps from post fixation to polymerization of resin blocks were carried out in a microwave oven (Pelco BioWave 34770, Pelco International, Redding, CA). Ultrathin sections were cut with a diamond knife (Reichert Ultracut E, Leica Inc., Wetzlar, Germany), stained with uranyl acetate and lead citrate and then examined by transmission electron microscopy (JEM-1011, JEOL USA Corp., Peabody, MA) at 75 kV. Digital electron micrographs were acquired directly with a 1024 × 1024 pixels CCD camera system (AMT Corp., Denver, MA).

### Statistics

Results are expressed as means ± SEM. N represents the number of individual experiments. Replicates within experiments are expressed as a mean for a single experiment. ANOVA and unpaired Student's *t*-test were conducted using InStat3 (GraphPad, San Diego, CA). Means were compared using ANOVA and Tukey's post-hoc test.

## Results

### AIEC infection decreases TER in T84 and MDCK-I epithelial cell monolayers

**Figure 1:**
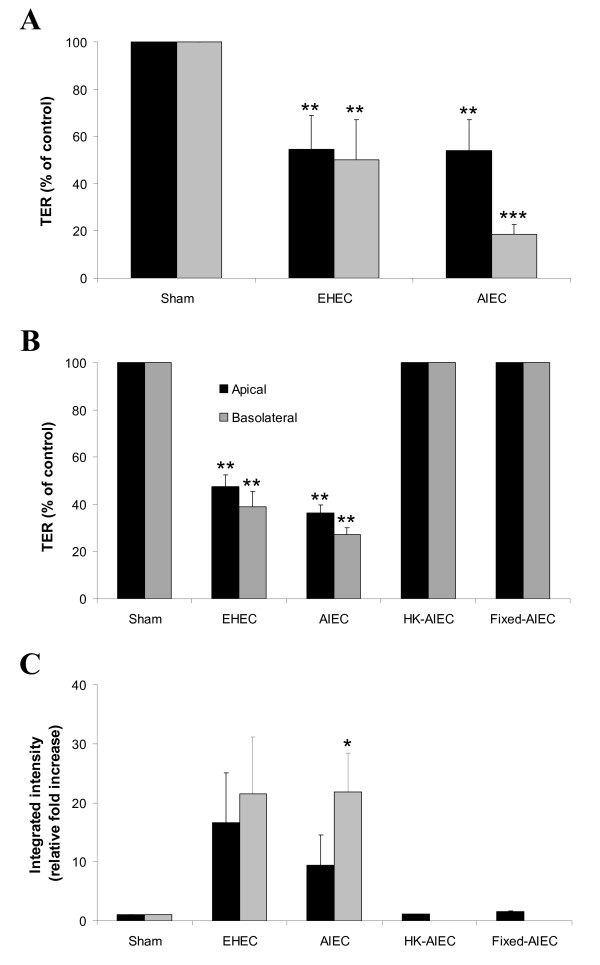
**AIEC, strain LF82 disrupts the integrity of polarized epithelial monolayers**. Model epithelial cell monolayers [T84 (**Panel A**) and MDCK-I (**Panels B & C**)] grown in Transwells were infected with either *E. coli*, strain LF82 (AIEC) or EHEC O157:H7 – employed as a positive control – for 16 h at 37°C. Both apical (black bar histograms) and basolateral (gray bars) infections of human intestinal T84 monolayers caused a reduction in TER (**Panel A**; N = 4–6). Similar effects of infection on monolayer integrity were observed when MDCK-I cell monolayers were infected with AIEC, strain LF82 (**Panel B**), together with an increase in permeability to a macromolecular (10-kilodalton) dextran probe, indicating barrier disruption (**Panels C**; N = 2–4). HK denotes heat-killed bacteria. ANOVA: * p < 0.05; ** p < 0.01; *** p < 0.001.

Similar to EHEC O157:H7, apical infection for 16 h with AIEC, strain LF82 caused a 46% reduction in TER in human colonic T84 cells (Figure 1A; ANOVA: p < 0.01, compared with uninfected sham controls). When the pathogen was introduced into the basolateral aspect of monolayers there was an 81% reduction in TER, relative to sham control monolayers, with AIEC infection (p < 0.001), compared to a 50% reduction with EHEC infection (p < 0.01; *t *test of AIEC vs. EHEC: p = 0.052). In contrast, both apical and basolateral infection of T84 monolayers with non-pathogenic *E. coli*, strain HB101 did not lead to a reduction in TER (N = 2).

Apical and basolateral infections of canine kidney-derived MDCK-I polarized monolayers with EHEC and AIEC caused a comparable reduction of 53–73% in TER (Figure 1B; ANOVA: p < 0.01). Live bacteria were required, because there was no drop in TER with either heat-inactivated or formaldehyde-fixed bacteria (Figure 1B). The effects were not due to the metabolic activity of bacteria on epithelial cells, since incubation with tissue culture medium corrected to pH 6 (the pH of medium after 16 h of infection) did not reduce TER (N = 2).

### Macromolecular permeability increases following AIEC infection of MDCK-I monolayers

**Figure 2:**
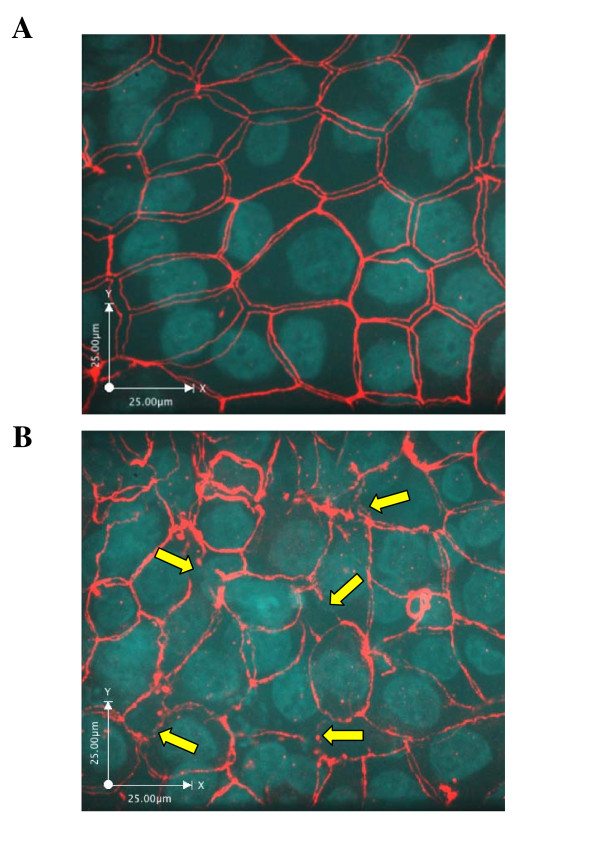
**Epithelial tight junctions are disrupted by AIEC infection**. MDCK-I monolayers were grown to confluence on 6.5 mm diameter Transwells and then either left uninfected (sham control; **Panel A**) or infected with AIEC, strain LF82 (**Panel B**) at a MOI of 100:1 for 16 h. Monolayers were then washed with PBS and fixed, blocked and incubated with primary rabbit anti-ZO-1 and the appropriate secondary antibody and DAPI. **Panel A**: Sham control cells showed a normal distribution of ZO-1, outlining the intercellular tight junctions. **Panel B**: AIEC infection resulted in disruption of ZO-1 localization with large gaps between cells (arrows). Approximate original magnifications: × 630.

Transcytosis of a 10-kDa dextran probe across monolayers supported the TER results. Consistent with previous reports [26], EHEC O157:H7 caused a dramatic increase in permeability to dextran, indicating breakdown of the epithelial barrier. Infection with AIEC also resulted in increased dextran permeability in MDCK-I cells (ANOVA: p < 0.05 for basolateral AIEC infection) comparable to findings seen with EHEC infection (Figure 1C; p > 0.05). There was a similar, but more modest, increase in permeability of T84 monolayers infected with AIEC (data not shown). There was no difference in permeability between apical and basolateral bacterial infections of monolayers of both cell types. Increases in permeability were not the result of epithelial cell death, since cells were still present in monolayers after 16 h of infection (Figure 2B).

### AIEC infection alters the distribution of ZO-1

Sham control MDCK-I cells (Figure 2A) demonstrated a normal distribution of ZO-1, delineating intact apical cellular junction complexes [27]. Consistent with effects on permeability, 16 h infection of MDCK-I monolayers with AIEC, strain LF82 (Figure 2B) led to profound disruption of ZO-1 with large gaps between cells with punctate and interrupted distribution of ZO-1, indicating disruption of this integral tight junction protein [28]. Nevertheless, cells in the monolayer remained viable, as demonstrated by the presence of nuclei and maintenance of normal cells shape and morphology.

### Disruption of MDCK-I monolayers is accompanied by AIEC invasion and bacterial replication

**Figure 3:**
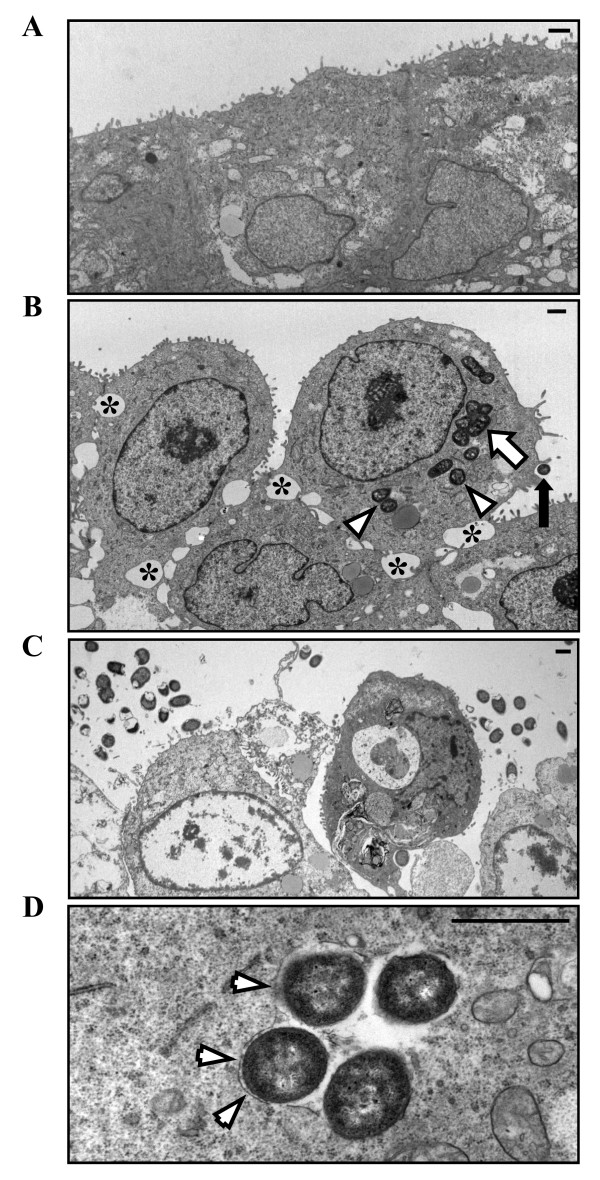
**AIEC disrupts MDCK-I monolayers and replicates in the cell cytoplasm**. MDCK-I monolayers were either left uninfected (sham control; **Panel A**) or infected with AIEC for 4 h (**Panel B & D**) and 48 h (**Panel C**). While uninfected cells maintained normal intercellular spaces (**Panel A**), transmission electron photomicrographs demonstrated disruptions in intercellular junctions between epithelial cells (*), as well as adhesion (black arrow) and invasion and replication (arrowheads and white arrow, respectively) of bacteria in 4 h AIEC, strain LF82-infected MDCK-I cells (**Panel B**). After 48 h of bacterial infection, monolayers were severely disrupted, accompanied by morphological changes within cells (**Panel C**). Some of the invasive bacteria appeared within membrane-bound vacuoles after 4 h of infection (arrowheads in **Panel D**). Measurement bar = 1 μ.

Transmission electron microscopy of infected MDCK-I monolayers was used to define the effect of AIEC infection of polarized monolayers. In contrast to sham control epithelial monolayers, which demonstrated tightly placed cells without expanded intercellular spaces (Figure 3A), AIEC-infected MDCK-I monolayers were disordered after 4 h of incubation, with spaces evident between adjacent cells and disruption of intercellular spaces. Loss of cellular polarity was also observed, as demonstrated by presence of microvilli on the lateral aspect of infected cells. Furthermore, consistent with previous reports [29], multiple bacteria were seen within cells 4 h after infection with effective replication, indicating that these organisms survive within the cytoplasm of epithelial cells (Figure 3B). Extension of bacterial infection to 48 h resulted in profound disruption of the monolayer, with complete separation between cells and terminal changes in cells, including loss of membrane integrity, chromatin condensation and ballooning of mitochondria (Figure 3C). This effect may be the result of bacterial overgrowth after 48 h of infection.

### Invasive AIEC are found within a membrane-bound, LAMP1 positive intracellular compartment

The ability of invasive microbes to survive in cells is dependent on creating a protective niche for replication [30]. Invasive AIEC were found in membrane-bound compartments 4 h after infection (Figure 3D). Presence of multiple organisms in one compartment suggests that they can effectively replicate within these vacuoles. Since the membrane appeared to be partially missing, it is possible that bacteria were escaping the vacuole.

**Figure 4:**
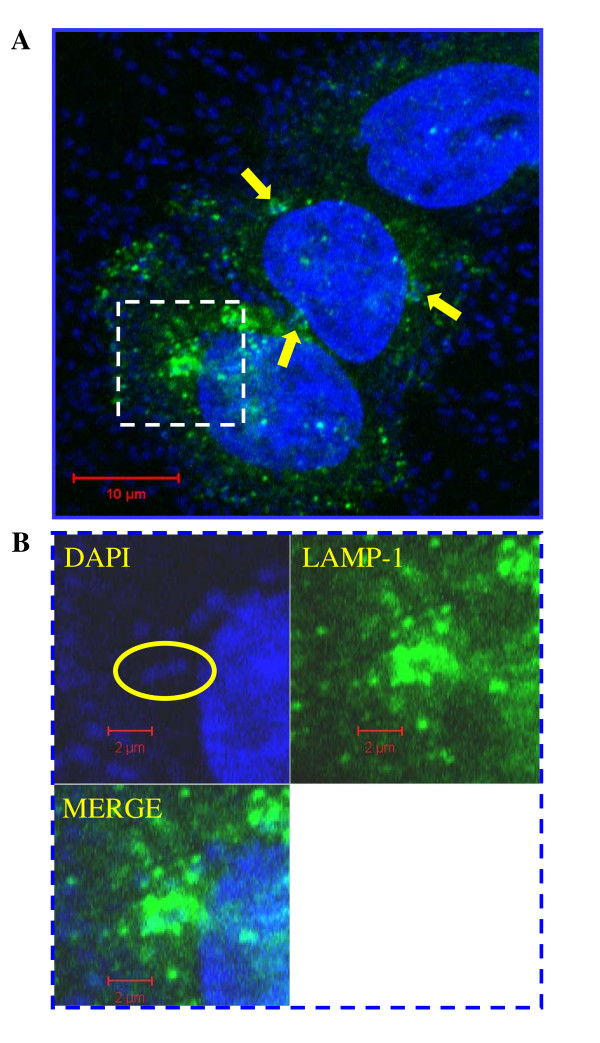
**AIEC localizes with late endosomes in infected epithelial cells**. Intestine 407 cells were infected with AIEC for 4 h and then fixed and stained with anti-LAMP1 antibody and DAPI. Multiple bacteria were observed adherent to cells and several invasive organisms (stained by DAPI) were found within the perinuclear region of the epithelial cell in LAMP1 positive compartments (arrows in **Panel A**). **Panel B**: enlarged image of dashed insert in **Panel A**, highlights colocalization of an invasive organism with the late endosomal marker LAMP1.

Confocal microscopy of infected intestine 407 cells, using an antibody against the late endosomal marker LAMP1, demonstrated that AIEC co-localized with this marker after 4 h of infection, indicating that vacuoles containing invasive AIEC were directed to the endosomal pathway in epithelial cells (Figure 4).

## Discussion

The intestinal barrier is comprised of a single layer of polarized epithelial cells serving to separate the luminal content, including microbes, from the underlying mucosa. Breaches in the epithelial barrier integrity result in penetration of luminal antigens and microbes, which stimulate pro-inflammatory responses, leading to chronic intestinal and systemic diseases, including IBD [1]. The importance of barrier maintenance in IBD is further highlighted by the development of colitis in mice expressing constitutively active myosin light chain kinase, which is involved in regulating the epithelial barrier [31]. AJCs are common targets of bacterial virulence, as displayed by multiple infection models affecting the integrity of the epithelial barrier [27]. Targeting of AJCs can be mediated by either bacterial toxins or effectors, by direct contact of the pathogen and by indirect effects on signaling pathways involved in host regulation of junction-associated proteins.

Recent research related to microbes isolated from patients with Crohn disease highlight a role for adherent-invasive *E. coli *strains, including strain LF82, in the pathogenesis of IBD [12]. Studies have focused on bacterial adhesion, invasion and replication in both epithelial cells and macrophages, as well as the accompanying inflammatory response [32]. For example, epithelial surface adhesions, such as CEACAMs, mediate attachment of various bacterial pathogens [33]. The association between LF82 and Crohn disease is linked to up-regulation of CEACAM5 and CEACAM6 by intestinal epithelial cells, which is induced by LF82 through TNF-α secretion [15]. Nevertheless, the ability of these microbes to disrupt the integrity of the epithelial barrier has not been extensively studied. Only a single published study describes AIEC-induced barrier disruption of Caco-2 cells [34]. Therefore, we employed transformed human colonic T84 cells and canine kidney MDCK-I cells as model polarized epithelia, which both express mature apical junctional complex proteins and maintain cell polarity [35], and are used extensively to study host-microbial interactions [22,36]. Furthermore, the utility of polarized epithelial monolayers in the study of AIEC infection was recently reported by Eaves-Pyles et al. [37] that demonstrated chemokine secretion by AIEC-infected Caco-2 and T84 monolayers leading to transmigration of immune cells.

Our findings indicate that infection of polarized monolayers with AIEC, strain LF82 leads to disruption of epithelial cell monolayers, as demonstrated by both reduced transepithelial electrical resistance and increased macromolecular permeability, as well as morphological defects in the structure of the AJCs of infected monolayers. The ability of invasive bacteria to disrupt monolayer integrity is described for some intestinal pathogens, such as *Shigella flexneri*, *Listeria monocytogenes *[38] and *Campylobacter jejuni *[39], while other bacteria, such as *Helicobacter pylori*, appear to alter AJCs without entering into the cytoplasm [28]. Since AIEC strains are associated with IBD, host cell invasion and barrier disruption, as presented in this study, are mechanisms that could contribute to intestinal injury and immune stimulation in affected patients.

Sasaki et al. [34] demonstrated the ability of LF82, as well as other AIEC strains, to reduce TER of Caco-2 monolayers and displace both ZO-1 and E-cadherin from AJCs. Our results confirm these findings in additional polarized epithelial cell lines and also reveal an increase in macromolecular permeability of infected monolayers. In addition, we show that introducing bacteria to the basolateral surface of T84 monolayers leads to a more profound reduction in TER. The significance of this finding is highlighted by the suggestion that other enteric pathogens, such as *C. jejuni*, enter epithelial cells through the basolateral membrane [40].

Our findings show that AIEC can replicate in membrane-bound vesicles, which positively stain with the late endosomal marker LAMP1. Similar to these findings, previous work suggests that strain LF82 is present in vacuoles in epithelial cells after invasion, but is also seen in the cytoplasm, suggesting that these bacteria can escape from the vacuoles [29]. Nevertheless, the phagocytic pathway involved in AIEC invasion of epithelial cells has not been characterized. Similar to our findings in epithelial cells, LF82 co-localizes with LAMP1 in infected macrophages [41], suggesting that there are common features in the intracellular fate of these microorganisms in different cell types.

The ability of AIEC to survive and replicate within the cytoplasm of epithelial cells is of relevance in IBD, since defects in the handling of intercellular microbes are considered to contribute to disease pathogenesis [11]. For example, absence of NOD2 in transgenic mice results in increased susceptibility to infection with intracellular pathogens, such as *Mycobacterium tuberculosis *[42]. Furthermore, the autophagy protein Atg16L1, which is also implicated in the pathogenesis of IBD [43], is involved in inflammatory responses to invasive microbes. Mice lacking Atg16L1 are more susceptible to chemically-induced colitis than wild-type animals subject of the same stress [44]. Therefore, it is plausible that defective handling of invasive AIEC strains in patients with IBD who have genetic mutations linked to defects in microbial processing contributes to intestinal injury, as suggested by increased response of monocytes from Crohn disease patients with NOD2 mutations to AIEC infection *in vitro *[45]. The findings of our study support the ability of AIEC to subvert one of the first lines of host innate defence, the epithelial cell barrier.

Taken together, these findings provide an improved understanding of mechanisms leading to intestinal injury and chronic immune stimulation by an AIEC bacterial strain that has been linked to IBD pathogenesis. Further insight into the mechanisms of epithelial barrier disruption and subversion of host defenses by intestinal pathogens is essential for developing novel strategies to interrupt the infectious process and thereby prevent its complications, including IBD.

## Conclusion

The invasive *E. coli *strain LF82, which is linked to IBD, disrupts AJCs of polarized epithelial monolayers and leads to increased macromolecular permeability and morphological interruption of intercellular tight junctions. After invasion into epithelial cells, the bacteria replicate within late endosomes. These findings contribute to current understanding of bacterial-mediated processes related to the pathogenesis of IBD and offer potential targets for intervening early in the course of the disease process.

## Abbreviations

AIEC: adherent-invasive *Escherichia coli*; AJCs: apical junctional complexes; CEACAM: carcinoembryonic antigen-related cell-adhesion molecules; DAPI: 4',6-diamidino-2-phenylindole; DMEM: Dulbecco's minimal essential medium; EHEC: Enterohemorrhagic *Escherichia coli*; FBS: fetal bovine serum; IBD: inflammatory bowel diseases; LAMP: lysosomal-associated membrane protein; MDCK: Madin-Darby canine kidney; MOI: multiplicity of infection; PBS: phosphate-buffered saline; TER: transepithelial electrical resistance; ZO-1: zonula occludens-1.

## Authors' contributions

EW designed and performed the experiments, analyzed the data and wrote the manuscript. JCO and SDG contributed to the discussion and data analysis. PMS designed the research and assisted in writing the manuscript. All authors read and approved the final manuscript.
